# Integration of virtual reality and eye-tracking-based feedback system improves rehabilitation observation skills

**DOI:** 10.3389/fresc.2026.1775722

**Published:** 2026-05-07

**Authors:** Kazuo Saito, Makoto Suzuki, Naoki Iso, Kilchoon Cho, Takuhiro Okabe, Takuya Matsumoto, Junichi Yamamoto

**Affiliations:** 1 Faculty of Health Sciences, Tokyo Kasei University, Sayama, Japan; 2 School of Health and Social Services, Saitama Prefectural University, Koshigaya, Japan; 3 Faculty of Medical Sciences, Shonan University of Medical Sciences, Yokohama, Japan; 4 Faculty of Systems Design, Tokyo Metropolitan University, Hachioji, Japan

**Keywords:** behavioral observation, expert therapists, eye-tracking, novice learners, occupational therapy education, single-case design, skill acquisition, virtual reality

## Abstract

**Background:**

Rehabilitation education has traditionally relied on an apprenticeship model, where the acquisition of practical skills, such as the ability to observe subtle patient movements to detect abnormalities, is highly dependent on the tacit knowledge of experienced practitioners. This lack of objective evaluation and standardized education presents a significant challenge. To address this, we integrated virtual reality (VR) and eye-tracking technologies to objectively measure and educate behavioral observation skills. This study aimed to quantitatively verify the educational effectiveness of this approach in novice therapists.

**Methods:**

We tested two hypotheses: first, that the integration of VR and eye-tracking can objectively quantify and differentiate the observation skills of expert and novice therapists and second, that feedback education based on this objective data can effectively improve novice skills to an expert level. Using a single-case, multiple-baseline design (A-B design), four novice physical therapy students observed a VR video of a patient with right-sided hemiparesis performing a reaching task. During the intervention, each novice received personalized feedback using their own visual scanning heatmaps compared to the expert average, along with detailed verbal explanations.

**Results:**

The novice baseline visual scanning patterns, which showed a concentrated focus on the patient's upper body, were quantitatively distinct from the broad, stable visual scanning patterns of four expert therapists. Through the intervention, data rate (PND) exceeded 70% in many participants and phases, indicating an immediate and powerful effect of the intervention. This improvement was sustained by one-month post-intervention. Concurrently, participants' self-efficacy, feasibility, and social validity scores showed notable increases and high median ratings.

**Discussion:**

These results provide the first scientific evidence that an integrated VR and eye-tracking system, offering data-driven feedback, can effectively and lastingly improve rehabilitation observation skills, which have previously been difficult to teach and evaluate.

## 1 Introduction

In the specific domain of neuromotor rehabilitation for stroke survivors, the ability to accurately observe movement is paramount. We define “behavioral observation skills” in this study not merely as visual tracking, but as the active attention process of identifying kinematic deviations (e.g., asymmetry in weight-bearing, insufficient joint excursion) to formulate clinical hypotheses. Experienced clinicians utilize a “professional visual scanning strategy,” characterized by efficient fixation on task-relevant areas (e.g., pelvis, knees) rather than salient but irrelevant features (1, 2). However, traditional apprenticeship models rely heavily on tacit knowledge, making it difficult to transfer this “visual search strategy” to novices. The critical gap this study addresses is determining whether explicit visualization of expert visual scanning patterns via VR feedback can effectively alter novice behavior and improve self-efficacy in a controlled Single-Case Experimental Design (A-B design).

While the integration of information and communication technologies (ICT) and the Internet of Things (IoT) has been attempted, their implementation in the complex environment of clinical practice has been limited, highlighting the urgent need for new educational tools that enable safe and systematic learning (3). Against this backdrop, virtual reality (VR) technology has recently garnered significant attention as a potentially transformative educational tool. VR can create immersive, lifelike environments, allowing for safe, repetitive experiential learning without placing patients at risk. This makes it particularly promising for the simulation of clinical observation and intervention techniques, where its educational efficacy is highly anticipated (4–7). Furthermore, previous studies have suggested that VR-based learning has the potential to objectively measure and enhance motor skills (8, 9). Despite this potential, the application of VR in rehabilitation education remains limited, and the rigorous scientific validation and standardization of its educational protocols are still in their early stages (10–12).

In parallel, eye-tracking technology has been widely recognized as a valuable tool for the objective assessment of clinical competence. Numerous studies have reported that experts allocate their attention efficiently to task-relevant areas, whereas novices exhibit unstable and inefficient visual scanning patterns (13–17). This finding is consistently observed across diverse professional fields, including surgery, sports, and radiology, strongly suggesting that eye-tracking data can serve as a reliable, objective indicator of professional skill. Moreover, “visual scanning-guided learning,” which involves presenting an expert's visual scanning pattern to a novice, has been proven to enhance learning outcomes in domains such as sports and surgical procedures (18). This approach holds promise for making an expert's thought process visible and for efficiently transforming a learner's cognitive strategies.

To address the limitations of traditional subjective methods, this study adopts a novel approach by integrating VR with eye-tracking technology. We hypothesized that feedback based on this objective data would visualize the expert's “visual scanning,” which is difficult to convey through traditional methods, and thereby lead to a highly effective educational outcome, ultimately addressing the long-standing challenge of tacit-knowledge dependency in traditional education. Building upon these insights, the present study combined VR with eye-tracking technology to enable the objective measurement of “behavioral observation skills,” which have historically been challenging to quantify and teach. By leveraging technology to provide objective, data-driven feedback, we proposed an effective educational method to address the long-standing challenge of tacit-knowledge dependency in traditional education. The primary objective was to quantitatively verify the educational effect of this approach on improving the observation skills of novices. To achieve this, we formulated and tested the following two hypotheses: (1) the observation skills of expert and novice therapists can be objectively quantified and distinguished by measuring their eye movements in a VR environment. (2) feedback education based on the objective data from hypothesis (1) can effectively improve the observation skills of novice therapists to an expert level.

## 2 Materials and methods

### 2.1 Study design and ethical considerations

This study adopted a multiple-baseline design as a single-case experimental design (19). The multiple-baseline design used in this study is a research design in which the number of baseline measurements differs across participants. By staggering the timing of the intervention across participants, this design allows the effects of repeated measurement and the passage of time to be controlled. Therefore, this research design was adopted in the present study to more rigorously examine the effects of the intervention. This research design is well-suited for rigorously evaluating the effects of an intervention on a small number of participants and offers high validity for assessing the efficacy of an individualized educational intervention (19, 20). The study was approved by the Research Ethics Committee of Tokyo Kasei University (Approval No.: SKE2022-03) and was conducted in accordance with the principles of the Declaration of Helsinki. All participants were thoroughly informed about the study's purpose and procedures and provided written informed consent.

### 2.2 Participants

Visual acuity was confirmed to be normal or corrected-to-normal vision based on participants' self-reports. Participants were recruited based on their clinical experience in the rehabilitation field, creating an expert group and a novice group.

The expert group consisted of four occupational therapists (three male, one female; mean age ± standard deviation: 47 ± 8.2 years (range: 39–55 years); clinical experience: 18.0 ± 5.1 years (range: 13–23 years). The inclusion criterion for the experts was a minimum of 10 years of clinical experience as an occupational therapist. This criterion was chosen to ensure not only long tenure but also the establishment of a “professional visual scanning strategy”—a specialized perspective for interpreting patient movements in relation to their underlying pathophysiology. While the expert group was more heterogeneous in age and background compared to the novices, this diversity was intended to construct a robust “Target Heatmap” that represents a generalized professional visual scanning strategy, minimizing the influence of any single expert's idiosyncratic habits. Conversely, the novice group was kept homogeneous to control for confounding variables within the single-case experimental design.

The novice group comprised four female students (age range: 21–22 years) enrolled in an occupational therapy program at a four-year university. The inclusion criterion for novices was the completion of their mandatory clinical practicum, ensuring they had foundational experience in behavioral observation. Although the novice group was limited to female students from a specific university, this homogeneous composition was appropriate for the purpose of a single-case study design, which aims to rigorously evaluate the intervention effect on individual participants.

### 2.3 Observation task and equipment

Participants performed an observation task by watching a 15-second VR video of a patient with right-sided hemiparesis performing a seated reaching task. The video was pre-recorded for this study and showed a therapist assisting a patient with seated exercises. The sequence included the therapist instructing the patient to reach to the right, the patient extending their left arm toward the target, losing balance, and being assisted by the therapist. The observation task was divided into the following three temporal phases: (1) Instruction Phase (0–4 s), during which the therapist instructs the patient on the movement; (2) Reach Phase (4–9 s), during which the patient reaches for the target with their left arm; and (3) Assist Phase (9–15 s), during which the patient loses balance and the therapist provides support to their trunk.

Visual scanning behavior was evaluated using a head-mounted display (HTC VIVE XR Elite, HTC Corporation) equipped with an infrared eye-tracking system (spatial accuracy: 0.5°–1.1°). This system allowed us to objectively measure the participants' visual scanning behavior. The headset features a resolution of 1,920 × 1,920 pixels per eye and a 110-degree field of view. Eye-tracking data were accessed via the SRanipal SDK (HTC Corporation). Before the experiment, a successful 5-point calibration procedure within the VR headset verified that all participants possessed sufficient visual function to track targets and distinguish the specific anatomical landmarks in the virtual environment necessary for the observational task. During the experiment, the video was displayed on the screen, and the position of the participant's point-of-regard was recorded at a 30 Hz sampling rate using the built-in infrared sensors. The data recording rate was limited to 30 Hz due to the system's prioritization of real-time VR rendering and feedback generation.

### 2.4 Eye-Tracking data evaluations

In this study, raw eye-tracking data were collected as continuous point-of-regard coordinates relative to the VR scene, and these continuous data were directly utilized to generate heatmaps representing visual attention. From the video, 30 anatomical landmarks were pre-set using a proprietary AI-based prediction system (VR Motion Detector, Next-System, Fukuoka, Japan). These landmarks included key anatomical points such as the forehead, nasal root, nasal tip, chin, sternum, bilateral ears, bilateral eyes, bilateral shoulders, bilateral elbows, bilateral wrists, bilateral middle finger metacarpophalangeal joints, bilateral middle finger fingertips, bilateral index fingertips, central pelvic region, bilateral hips, bilateral knees, bilateral ankles, and bilateral toe tips. The use of this AI enabled the instantaneous and objective identification of landmark positions even in a dynamic clinical setting, thereby ensuring the reliability of the eye-tracking data for the observation task.

By capturing participants' eye-tracking data during the observation of rehabilitation movements in a VR space, we objectively evaluated the differences in information-gathering patterns between experts and novices. The area of visual attention was defined as the region containing a visual attention probability density of 37.5% or higher relative to the peak. We then calculated the number of bodily landmarks contained within this defined region.

These were aggregated separately for the upper and lower limbs. This quantitative metric was highly effective for intuitively understanding the differences in attention allocation between novices and experts. By combining the qualitative information from the heatmaps with the quantitative data on the number of skeletal points observed, we were able to evaluate not only the location of visual attention but also the depth and importance of the attention paid to different body parts.

During the experiment, the raw eye-tracking data were stored locally as CSV files via a custom Unity script for offline analysis. For data quality control and filtering, this study relied on the built-in algorithms provided by the HTC SRanipal SDK. The SDK automatically handles blink detection and compensates for minor tracking losses in real-time. Specifically, the raw eye movement coordinates were recorded only when the SDK returned a “valid” tracking status for the eyes, which inherently filtered out blinks and severe measurement artifacts. Therefore, no additional offline smoothing filters were applied to the continuous point-of-regard data. This SDK-based data quality control protocol is an established method that has been widely utilized in recent eye-tracking studies (21–23).

### 2.5 Experimental design and procedure

Before the study began, the four participants in the expert group observed the same VR video five times. Their eye-tracking data were recorded and evaluated to serve as the reference standard for the educational intervention with the novice group.

**Figure 1:**
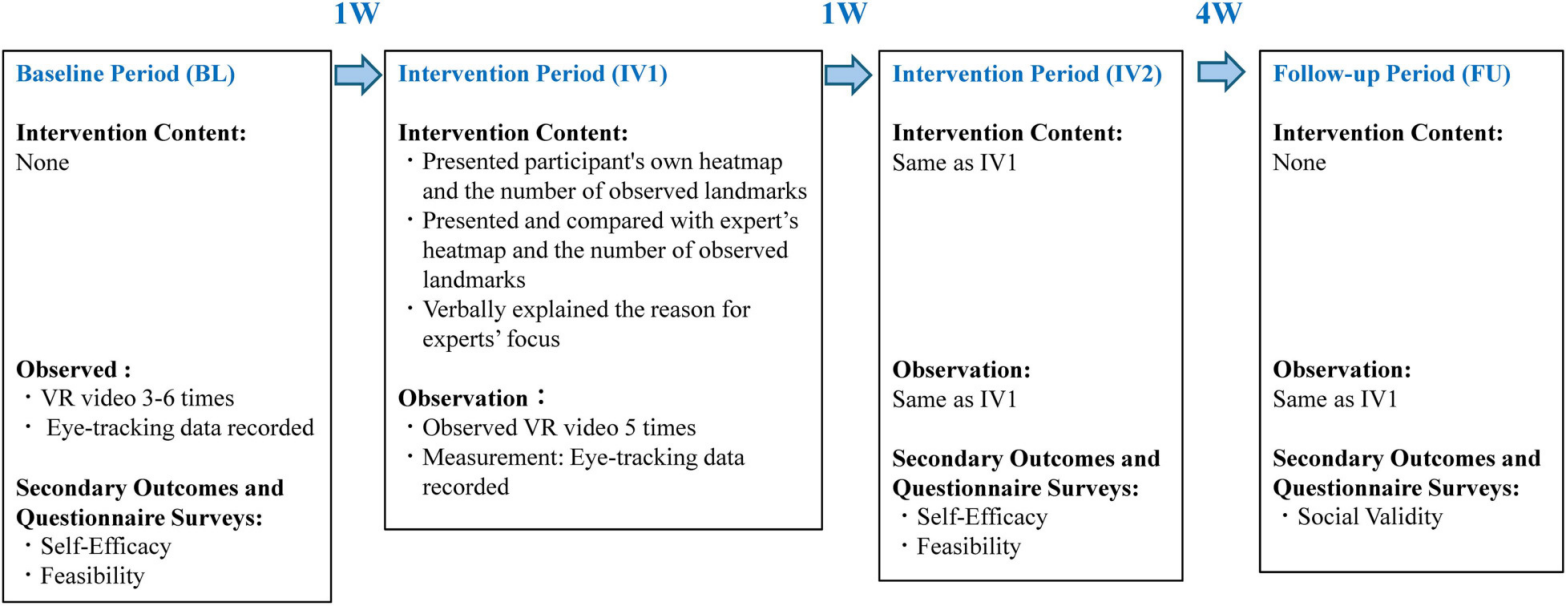
The experimental design. The outlines the four phases of the study: Baseline Period (BL), Intervention Period 1 (IV1), Intervention Period 2 (IV2), and Follow-up Period (FU). It also describes the intervention content and observations for each period.

For the novice group, we adopted a single-case experimental design with a multiple-baseline design (A-B design). The procedure consisted of four phases: a baseline phase, two intervention phases, and a follow-up phase (Figure 1).

The first phase was the Baseline Phase (BL), where each of the four novice participants watched the same VR video three to six times, and their eye-tracking data were recorded to establish their baseline observation patterns.

Following the baseline, a 20-minute, individually optimized feedback session was conducted. For the purpose of the educational feedback session, the participant's heatmap and eye-tracking data from the final baseline trial were consistently utilized every time and compared against the expert target to highlight immediate discrepancies. However, for the statistical analysis of intervention effects (LLT model), all data points collected during the baseline phase were utilized to estimate the trend. A detailed verbal explanation was also provided by the primary researcher (KS), an experienced physical therapist, on why the experts focused on specific body parts in each scene. For example, during the Instruction Phase, the expert explained the patient's right-sided trunk tilt due to hemiparesis and seated balance. In the Reach Phase, the expert explained the lack of movement in the right upper and lower limbs and the insufficient weight shift. For the Assist Phase, the expert emphasized observing the patient losing balance and the therapist's support. This process provided tailored instruction based on each participant's specific characteristics.

Based on this educational protocol, an observational session was conducted five times after receiving education based on data from experts. This served as Intervention Phase 1 (IV1). Intervention Phase 2 (IV2) was conducted to promote learning retention and consolidate skill acquisition. In this phase, participants performed the observation tasks without receiving the educational feedback session again, relying instead on the skills acquired prior to IV1.

Finally, a Follow-up Phase (FU) was set one month after the completion of IV2. Participants watched the VR video five more times without any intervention to evaluate the maintenance of the learned observation skills. The one-month period was chosen as it is appropriate for assessing long-term skill retention rather than just a temporary effect.

### 2.6 Secondary outcomes and questionnaire surveys

In addition to changes in eye-tracking data, we also conducted questionnaire surveys to evaluate the psychological changes resulting from the intervention (Figure 1). Three types of questionnaires were used: self-efficacy, feasibility, and social validity. First, self-efficacy was evaluated at the end of the baseline phase and Intervention Phase 2 (Table 1). Second, feasibility was evaluated at the end of Intervention Phase 2 (Table 2). Third, social validity was evaluated during the follow-up phase (Table 3). We assessed the potential for future application of VR training in clinical practice and the depth of understanding gained compared to traditional learning methods. By measuring these psychological outcomes, we were able to comprehensively understand not only the improvement in technical skills but also the learners' intrinsic motivation and their subjective evaluation of the educational system itself.

**Table 1:** Self-Efficacy questionnaire.

Characteristics	Value
A. General Self-Efficacy Questionnaire (4-point Likert Scale)	
Question 1	To what extent do you believe you can achieve goals you set for yourself?
Question 2	To what extent do you believe you can achieve good results with some ingenuity and effort?
Question 3	To what extent do you believe you can perform well even with different tasks?
Question 4	To what extent do you believe you can perform well even with challenging tasks?
B. Task-Specific Self-Efficacy Questionnaire (Yes or No).	
Question 1	Do you believe you will succeed in the next observation?
Question 2	Do you believe you can perform well in the next observation, even if it is a challenging task?
Question 3	Do you believe you can achieve the goals you set for yourself in the next observation?
Question 4	Do you believe you can achieve good results in the next observation through ingenuity and effort?

**Table 2:** Feasibility questionnaire (7-point Likert scale).

Item	Question
Question 1	Is VR-based visual attention training effective?
Question 2	Is VR-based visual attention training useful in clinical practice?
Question 3	Is VR-based technical training effective in clinical practice?
Question 4	What are the potential areas for improvement in VR-based visual attention training?

**Table 3:** Social validity questionnaire (7-point Likert scale).

Item	Question
Question 1	VR-based visual scanning training seems applicable to the future work of occupational therapists.
Question 2	Learning through VR-based instruction provided a deeper understanding compared to traditional teaching methods.

### 2.7 Statistical analysis

A series of analyses was conducted to evaluate changes in visual scanning behavior and educational effects. First, to facilitate a direct comparison between the novices' and experts' visual scanning patterns, we normalized the data. This was accomplished by subtracting the mean number of body landmarks observed by the four expert therapists from the number of body landmarks observed by each novice. This difference data serves as an indicator of the degree to which the participants' visual scanning deviates from that of the experts.

Next, a Local Linear Trend (LLT) model was applied to the normalized difference data across the baseline and Intervention 1 phases, assuming that both the level and slope components followed Gaussian random walks (20). The model is defined by the following state-space equations:

$$y_{t}=\alpha_{t}+\varepsilon_{t}\;(\mathrm{Observation}\;\mathrm{Equation})$$

$$\alpha_{t+1}=\alpha_{t}+\beta_{t}+\xi_{t}\;(\mathrm{Level}\;\mathrm{Equation})$$

$$\beta_{t+1}=\beta_{t}+\zeta_{t}\;(\mathrm{Slope}\;\mathrm{Equation})$$

where $\alpha_{t}$ is the level, $\beta_{t}$ is the slope, $y_{t}$ is the normalized difference data, $\varepsilon_{t}$ is the random variable, $\xi_{t}$ is the disturbance at the level, and $\zeta_{t}$ is the disturbance at the slope. This model was employed to estimate the state values for both phases and to predict the forecast values for the Intervention 1 phase by extrapolating the baseline level and trend. Finally, the estimated state and forecast values were linearly interpolated to generate 100 equally spaced data points over the same time span, enabling a more continuous and interpretable comparison of the observed trajectories.

Finally, the Percentage of Non-overlapping Data (PND) was computed using the upsampled data to evaluate the effect of the intervention, defined as the proportion of state values during the intervention phase that surpassed the corresponding forecasted values. A PND of 70% or greater was considered indicative of a meaningful intervention effect (20, 24). This sophisticated modeling approach was chosen to minimize the influence of noise inherent in small-sample time-series data and to more accurately capture the underlying trajectory of change following the intervention.

## 3 Results

### 3.1 Expert visual scanning behavior

The average ± standard number of skeletal points recorded across the phases, which quantified the experts' visual scanning behavior, was subsequently set as the target for novice feedback education. The specific visual scanning metrics for the experts across the three phases (Instruction, Reach, and Assist) are summarized in Table 4. The standard deviations for the observed body landmarks were consistently low (less than ± 1.5 in the total body count for all phases, as shown in the ± 0.96 to ± 3.11 range for the total points), indicating a high consistency and stability in the visual scanning patterns among the four experts. These mean and standard deviation data confirmed a shared “professional visual scanning strategy.” The mean values were then used to create a unified expert average heatmap, which served as the objective target for the novices during the personalized feedback education.

**Table 4:** Expert visual scanning behavior metrics (mean ± SD).

Task Phase	Total Skeletal Points	Upper Body Points	Lower Body Points
Instruction	23.3 ± 1.0	18.0 ± 0.8	5.3 ± 0.5
Reach	25.0 ± 1.4	20.8 ± 1.0	4.3 ± 1.0
Assist	21.5 ± 3.1	15.3 ± 2.2	6.3 ± 1.0

Data represent the average number of skeletal points observed within the high-density visual attention area.

### 3.2 Novice visual scanning behavior changes

Changes in the normalized number of body landmarks observed within high-density visual attention areas for each novice participant were quantitatively analyzed across the different phases.

**Figure 2:**
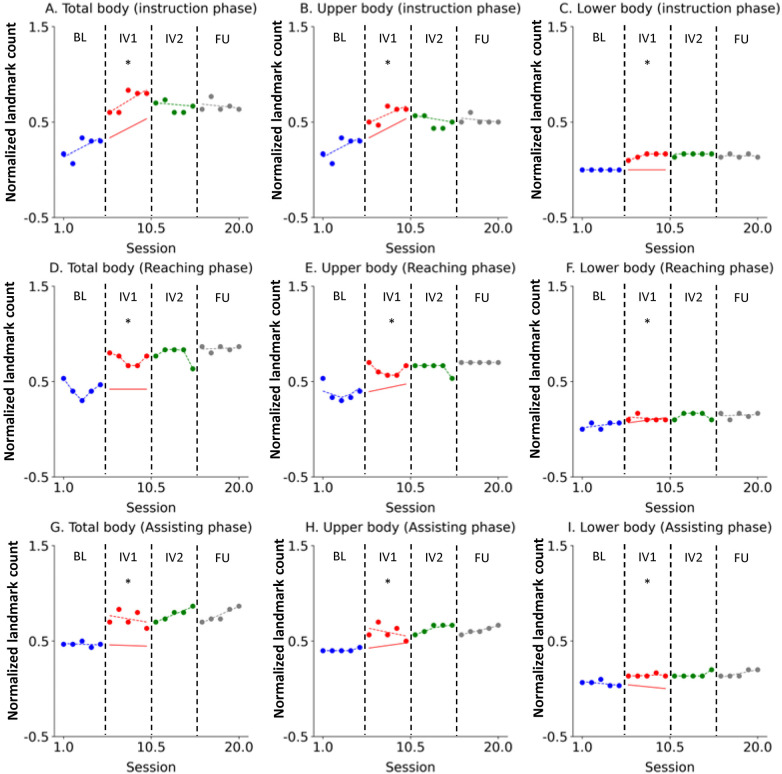
Novice 1 visual scanning behavior changes across study phases. The data are presented in separate graphs categorized by task phase (Instruction, Reaching, Assisting) and body region (Total body, Upper body, Lower body). Actual data points are represented by dots: blue for the Baseline (BL) phase, red for Intervention 1 (IV1), green for Intervention 2 (IV2), and gray for the Follow-Up (FU) phase. State values are indicated by dashed lines using the corresponding color scheme (blue, red, green, and gray). The forecast value for the IV1 phase is represented by a red solid line. An asterisk (*) denotes that the Percentage of Non-overlapping Data (PND) was greater than 70%. **(A)** Total body (instruction phase), **(B)** Upper body (instruction phase), **(C)** Lower body (instruction phase), **(D)** Total body (Reaching phase), **(E)** Upper body (Reaching phase), **(F)** Lower body (Reaching phase), **(G)** Total body (Assisting phase), **(H)** Upper body (Assisting phase), **(I)** Lower body (Assisting phase).

For Novice 1, the number of body landmarks observed within high-density visual attention areas for the total body, upper body, and lower body showed an immediate and strong increase upon the commencement of IV1 (Figure 2). This improvement was maintained and, in some cases, reinforced throughout IV2. The acquired broad visual scanning patterns were well-sustained one month later in the FU phase, confirming the durability of the learning effect.

**Figure 3:**
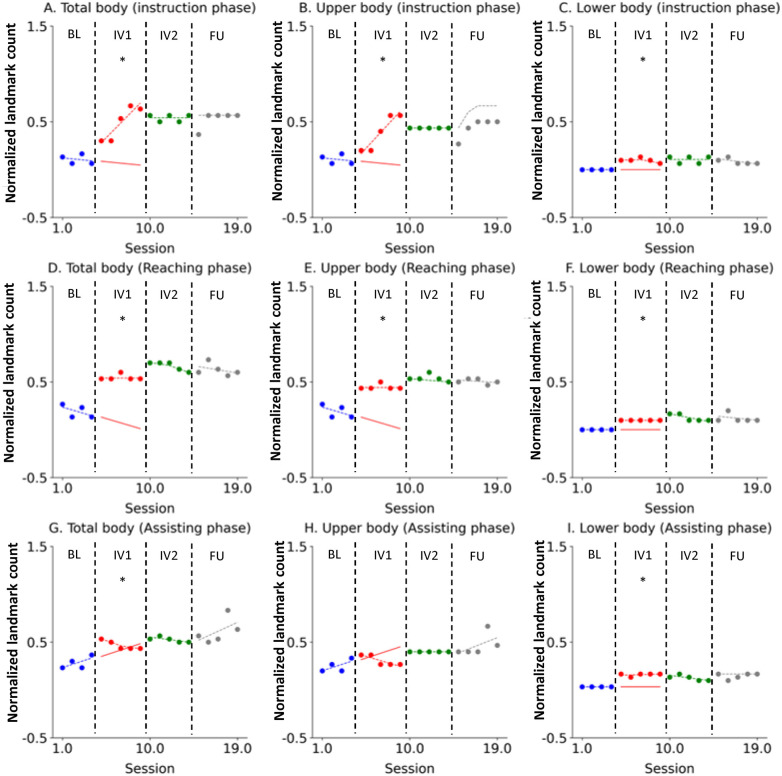
Novice 2 visual scanning behavior changes across study phases. The data are presented in separate graphs categorized by task phase (Instruction, Reaching, Assisting) and body region (Total body, Upper body, Lower body). Actual data points are represented by dots: blue for the Baseline (BL) phase, red for Intervention 1 (IV1), green for Intervention 2 (IV2), and gray for the Follow-Up (FU) phase. State values are indicated by dashed lines using the corresponding color scheme (blue, red, green, and gray). The forecast value for the IV1 phase is represented by a red solid line. An asterisk (*) denotes that the Percentage of Non-overlapping Data (PND) was greater than 70%. **(A)** Total body (instruction phase), **(B)** Upper body (instruction phase), **(C)** Lower body (instruction phase), **(D)** Total body (Reaching phase), **(E)** Upper body (Reaching phase), **(F)** Lower body (Reaching phase), **(G)** Total body (Assisting phase), **(H)** Upper body (Assisting phase), **(I)** Lower body (Assisting phase).

Similarly, for Novice 2, a strong and immediate intervention effect was observed across most body regions (Figure 3). As with Novice 1, this change was maintained and reinforced throughout IV2, and the acquired broad visual scanning patterns were well-sustained one month later in the FU, confirming the durability of the learning effect.

**Figure 4:**
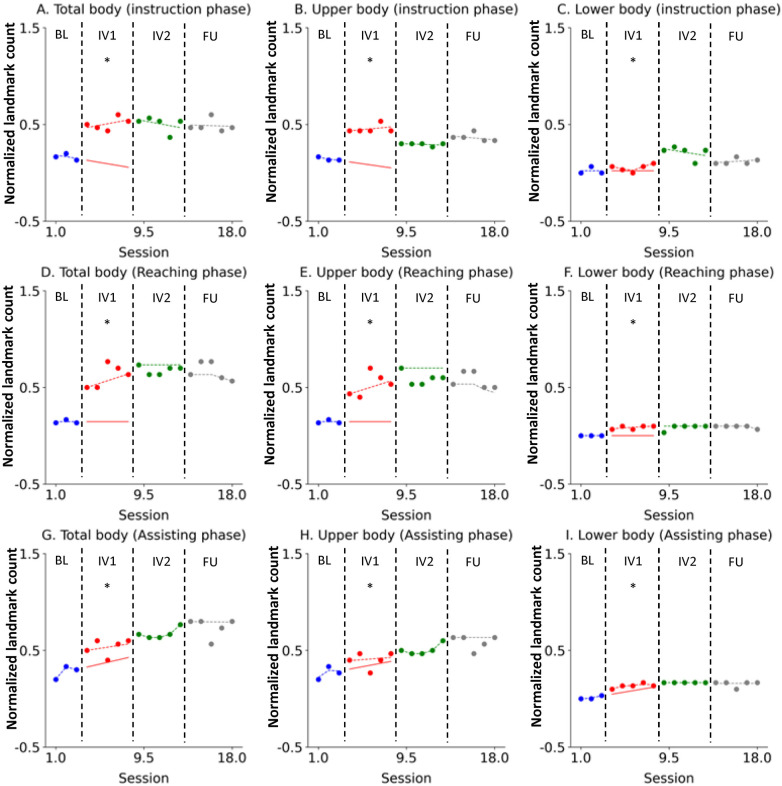
Novice 3 visual scanning behavior changes across study phases. The data are presented in separate graphs categorized by task phase (Instruction, Reaching, Assisting) and body region (Total body, Upper body, Lower body). Actual data points are represented by dots: blue for the Baseline (BL) phase, red for Intervention 1 (IV1), green for Intervention 2 (IV2), and gray for the Follow-Up (FU) phase. State values are indicated by dashed lines using the corresponding color scheme (blue, red, green, and gray). The forecast value for the IV1 phase is represented by a red solid line. An asterisk (*) denotes that the Percentage of Non-overlapping Data (PND) was greater than 70%. **(A)** Total body (instruction phase), **(B)** Upper body (instruction phase), **(C)** Lower body (instruction phase), **(D)** Total body (Reaching phase), **(E)** Upper body (Reaching phase), **(F)** Lower body (Reaching phase), **(G)** Total body (Assisting phase), **(H)** Upper body (Assisting phase), **(I)** Lower body (Assisting phase).

Novice 3 exhibited consistent and comparable educational effects to the other participants (Figure 4). This positive change was maintained and reinforced throughout IV2, and the acquired broad visual scanning patterns were well-sustained one month later in the FU, confirming the durability of the learning effect.

**Figure 5:**
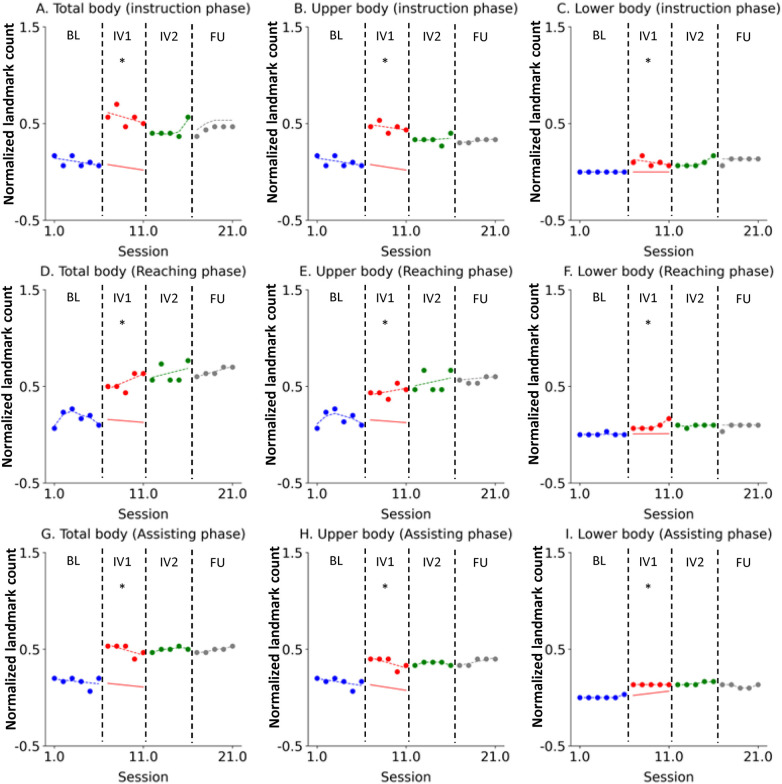
Novice 4 visual scanning behavior changes across study phases. The data are presented in separate graphs categorized by task phase (Instruction, Reaching, Assisting) and body region (Total body, Upper body, Lower body). Actual data points are represented by dots: blue for the Baseline (BL) phase, red for Intervention 1 (IV1), green for Intervention 2 (IV2), and gray for the Follow-Up (FU) phase. State values are indicated by dashed lines using the corresponding color scheme (blue, red, green, and gray). The forecast value for the IV1 phase is represented by a red solid line. An asterisk (*) denotes that the Percentage of Non-overlapping Data (PND) was greater than 70%. **(A)** Total body (instruction phase), **(B)** Upper body (instruction phase), **(C)** Lower body (instruction phase), **(D)** Total body (Reaching phase), **(E)** Upper body (Reaching phase), **(F)** Lower body (Reaching phase), **(G)** Total body (Assisting phase), **(H)** Upper body (Assisting phase), **(I)** Lower body (Assisting phase).

Finally, for Novice 4, the intervention had an immediate and strong effect on the total number of body landmarks observed (Figure 5). Specifically, the estimated level (*α*_t) for the Total body in all task phases showed a sharp upward shift from the end of the baseline, reflecting an immediate effect. Furthermore, the positive slope (*β*_t) throughout IV1 indicated a continuous learning trend that was sustained through subsequent phases. Improvements in visual scanning patterns on both the upper and lower body were evident and sustained throughout the IV2 and FU phases.

### 3.3 Evaluation of self-efficacy, feasibility, and social validity

In addition to changes in visual scanning behavior, psychological changes resulting from the intervention were also observed. First, an increase in self-efficacy was confirmed in all participants. In the General Self-Efficacy Questionnaire (4-point scale), the median score increased from 2.0 at baseline to 3.0 or 3.5 after the intervention across all four questions (Table 5A). Similarly, task-specific self-efficacy (Table 5B) also showed improvement across all questions; for instance, the percentage of participants who answered “Yes” to the question regarding performing well in a challenging task (Question 2) increased from 75% at baseline to 100% after the intervention. Similarly, task-specific self-efficacy (Yes/No) scores also improved, with the percentage of participants who answered “Yes” to Question 2 (“Do you believe you can perform well in the next observation, even if it is a challenging task?”) increasing from 75% at baseline to 100% after the intervention (Table 5B). It is noted, however, that the percentage of participants who answered “Yes” to Question 1 (“Do you believe you will succeed in the next observation?”) remained at 50% both before and after the intervention, suggesting a ceiling effect or a particularly challenging question.

**Table 5:** Comparison of self-efficacy scores before and after the intervention.

Question Item	BL	IV2
A. General Self-Efficacy Questionnaire		
Question 1	2.0 (0.0)	3.0 (0.0)
Question 2	2.0 (0.0)	3.5 (1.0)
Question 3	2.0 (1.5)	3.0 (0.8)
Question 4	2.0 (0.8)	3.0 (0.0)
B. Task-Specific Self-Efficacy Questionnaire.		
Question 1	50%	50%
Question 2	75%	100%
Question 3	25%	50%
Question 4	50%	75%

Values are presented as median (interquartile range).

Values indicate the percentage of participants who gave a “Yes” response.

Furthermore, in the feasibility questionnaire conducted at the end of IV2, all participants rated the VR training highly as “effective” and “clinically useful,” with median scores of 6.0 and 7.0, respectively. They also gave the highest possible median score of 7.0 for the effectiveness of VR-based technical training in a clinical setting. These results indicate that the participants themselves subjectively recognized the high educational value of this training (Table 6).

**Table 6:** Scores for the feasibility questionnaire.

Question	Score (Median [IQR])
Question 1	6.0 (0.8)
Question 2	7.0 (0.8)
Question 3	7.0 (0.0)

Values are presented as median (interquartile range).

Similarly, in the social validity survey conducted during the follow-up phase, participants gave high ratings (median of 6.0) for both the “potential for future clinical application” and the “depth of understanding compared to traditional learning methods” (Table 7).

**Table 7:** Scores for the social validity questionnaire.

Question	Score (Median [IQR])
Question 1	6.0 (0.8)
Question 2	6.0 (0.8)

Values are presented as median (interquartile range).

## 4 Discussion

The empirical findings obtained in this investigation substantiate the two hypotheses initially proposed. We demonstrated that we could objectively distinguish the observation skills of experts and novices by measuring their visual scanning behavior in a VR environment (Hypothesis 1), and that data-driven feedback education effectively improved novice skills (Hypothesis 2). This is the key novelty of our study: it shows that “behavioral observation skills”—which have historically depended on the instructor's experience—can be taught objectively and effectively using VR.

The first major contribution of this study is the application of VR-based eye-tracking to objectively capture and quantify rehabilitation observation skills, which have traditionally been difficult to measure. While previous studies have shown that expert clinicians and novice learners have different visual scanning patterns across various fields (13–17), our research is the first to combine eye-tracking with VR to analyze and visualize the specific diagnostic visual scanning patterns of expert occupational therapists in a dynamic patient scenario. This is a crucial difference from prior work, as previous research on visual scanning patterns often used static images or real-world tasks that lacked the controlled, reproducible environment of a VR simulation. The consistent tendency for novices to focus their visual search on the patient's upper body during the baseline phase was in clear contrast to the broad visual scanning pattern of the experts. This narrow focus on a limited area expanded to a broader, expert-like visual search pattern that included the lower body and the base of support after the data-driven educational intervention. This finding demonstrates that VR-based feedback effectively broadened the novices' visual scanning to encompass essential body parts, which had previously been overlooked. These observations re-confirm the difference in visual scanning patterns between experts and novices, further substantiating that eye-tracking data can serve as an objective indicator of clinical competence.

A second novel contribution is the demonstration that VR-based feedback education has an immediate and lasting learning effect. Prior research on visual scanning-guided learning has shown promise in fields like sports and surgery, where an expert's visual scanning pattern is visually presented to novices (18). Our study is the first to apply this concept to rehabilitation education, using personalized heatmaps and verbal explanations to transfer an expert's “diagnostic visual scanning” to a novice. This approach, which goes beyond simple visual imitation, effectively transformed the novices' cognitive strategies with explaining why the experts focused on specific areas. This analysis was strengthened by the use of the LLT model, which mitigated the influence of small-sample noise and baseline variability, thus increasing the reliability of our causal inference. The fact that the PND reached 100% in many cases provides quantitative proof of an extremely strong, immediate effect, signifying that every post-intervention data point showed improvement over all baseline data points. Furthermore, the retention of the acquired skills one month later confirms the durability of this learning effect.

A third, and equally important, contribution is the verification of the psychological impact of the educational system. Beyond the technical improvements, the accompanying increase in self-efficacy suggests a significant psychological effect of this educational intervention. Beyond the technical improvements, the accompanying increase in self-efficacy suggests a significant psychological effect of this educational intervention. For example, the percentage of participants who answered “Yes” to the question “Do you believe you can perform well in the next observation, even if it is a challenging task?” (Task-Specific Question 2) increased from 75% at baseline to 100% after the intervention. This outcome aligns with Bandura's theory of “mastery experience,” illustrating that the enhancement of technical skills and the growth of self-efficacy develop in tandem. As proposed by Bandura, self-efficacy is a primary predictor of behavioral change (25). The VR training in this study made the expert model visible and provided opportunities for participants to repeatedly experience success in a safe environment. This process likely enhanced the participants' feeling of “I can do this,” thereby promoting skill consolidation and motivation to practice (26). These results indicate a comprehensive effect of the educational intervention on both technical skills and psychological aspects.

The combination of personalized, data-driven feedback based on integrating VR with eye-tracking technology and an immersive VR environment enabled participants to efficiently acquire more specialized visual strategies, demonstrating both the immediate and sustained effects of the learning. The method established in this study holds promise as a scalable, individually optimized learning approach with the potential to advance clinical education in rehabilitation.

Previous research on medical expertise has extensively documented the cross-sectional differences between experts and novices. For instance, studies in radiology and sports have shown that experts employ a more efficient “visual search” strategy, focusing on relevant cues while ignoring redundant information (1, 2). However, these studies were primarily descriptive. Our study advances this field by moving from description to intervention. Unlike prior works that identified “what” experts see, we demonstrated “how” to transfer this expert strategy to novices. Furthermore, while video-based feedback has been used in surgical training (18), our approach integrates VR immersion and explicit heatmap feedback with detailed verbal explanations within a rehabilitation context. This comprehensive combination allows learners not just to mimic eye movements, but to understand the clinical reasoning (the “why”) behind the expert's visual scanning.

A limitation of this study is the sampling rate of 30 Hz. While insufficient for analyzing micro-saccade dynamics, previous research suggests that this resolution is adequate for heatmap-based educational feedback and analyzing dwell times on large Areas of Interest (AOIs) (27), which were the primary metrics used to facilitate novice learning in this study. Another limitation of this study is that participants were restricted to four female students from a specific university. To enhance the generalizability of these findings to a broader population of rehabilitation learners, future research should include a more diverse group of participants considering factors such as gender, specialization, and clinical experience. Furthermore, although the expert group intentionally included individuals with diverse backgrounds, the heterogeneity within the small sample size of only four experts may have influenced the target heatmap. In addition, future studies should also recruit a larger number of experts to further validate and generalize the professional visual scanning model. By integrating these insights, we hope to contribute to the creation of a next-generation clinical education system optimized for individual learners.

## 5 Conclusion

This study provides the first scientific evidence that integrating VR and eye-tracking-based feedback—modeled on expert therapists' visual scanning behavior—has significant educational value for teaching observation skills to novices. Using objective visual scanning metrics based on the number of skeletal points observed within high-density visual attention areas, we substantiated a significant improvement in novice visual scanning behavior following the intervention. These results provide objective evidence that a data-driven educational system can effectively address the long-standing challenge of teaching and evaluating subtle behavioral observation skills in rehabilitation education.

## Data Availability

The original contributions presented in the study are included in the article/Supplementary Material, further inquiries can be directed to the corresponding author.
